# Impact of pharmacist-conducted anticoagulation patient education and telephone follow-up on transitions of care: a randomized controlled trial

**DOI:** 10.1186/s12913-021-06156-2

**Published:** 2021-02-16

**Authors:** Lamis R. Karaoui, Elsy Ramia, Hanine Mansour, Nisrine Haddad, Nibal Chamoun

**Affiliations:** 1 Department of Pharmacy Practice, School of Pharmacy, Lebanese American University, P.O. Box: 36 (S23), Byblos, Lebanon; 2 Department of Pharmacy, American University of Beirut Medical Center, P.O.Box: 11 – 0236, Riad El Solh, Beirut, 1107 2020 Lebanon

**Keywords:** Anticoagulation, Discharge counseling, Transitions of care, Readmissions, Bleeding

## Abstract

**Background:**

There is limited published data in Lebanon evaluating the impact of supplemental education for anticoagulants use, especially DOACs, on clinical outcomes such as bleeding. The study aims to assess the impact of pharmacist-conducted anticoagulation education and follow-up on bleeding and readmission rates.

**Methods:**

This study was a randomized, non-blinded interventional study conducted between August 2017 and July 2019 in a tertiary care teaching Lebanese hospital. Participants were inpatients ≥18 years discharged on an oral anticoagulant for treatment. Block randomization was used. The control group received the standard nursing counseling while the intervention group additionally received pharmacy counseling. Phone call follow-ups were done on day 3 and 30 post-discharge. Primary outcomes included readmission rates and any bleeding event at day 3 and 30 post-discharge. Secondary outcomes included documented elements of education in the medical records and reported mortality upon day 30 post-discharge.

**Results:**

Two hundred patients were recruited in the study (100 patients in each study arm) with a mean age of 73.9 years. In the pharmacist-counseled group, more patients contacted their physician within 3 days (14% versus 4%; *p* = 0.010), received explicit elements of education (*p* < 0.001) and documentation in the chart was better (*p* < 0.05). In the standard of care group, patients were more aware of their next physician appointment date (52% versus 31%, *p* < 0.001). No difference in bleeding rates at day 3 and 30 post-discharge was observed between the groups.

**Conclusions:**

Although pharmacist-conducted anticoagulation education did not appear to reduce bleeding or readmission rates at day 30, pharmacist education significantly increased patient communication with their providers in the early days post-discharge.

**Trial registration:**

Lebanon Clinical Trial Registry LBCTR2020033424. Retrospectively registered. Date of registration: 06/03/2020.

**Supplementary Information:**

The online version contains supplementary material available at 10.1186/s12913-021-06156-2.

## Background

Oral anticoagulants (OACs) encompass traditional vitamin K antagonists (VKAs) as well as direct oral anticoagulants (DOACs). DOACs have broadened treatment options for stroke prevention in atrial fibrillation and treatment and prevention of thromboembolic diseases, which has led to a larger number of patients receiving adequate antithrombotic therapy [1–4]. With enhanced convenience, similar efficacy and significantly lower bleeding risk, as compared to vitamin K antagonists (VKAs), DOACs have become the preferred anticoagulant option [1, 2, 5]. Despite the widespread use of DOACs, VKAs remain a pillar in the armamentarium of anticoagulation for patients with specific conditions such as mechanical heart valves and severe renal impairment.

Anticoagulants are a major cause of acute and serious adverse drug events (ADEs) among hospitalized patients and older outpatients [6, 7]. Since 2008, the Joint Commission instituted the National Patient Safety Goal (NPSG.03.05.01) to reduce patient harm associated with anticoagulants [8]. In response to this goal, many hospitals instituted anticoagulation dosing protocols, emphasized patient counseling and even explored the effect of inpatient initiatives on post-discharge safety outcomes [9].

Ensuring effective care transitions for patients on OACs is imperative since the initiation and modification of anticoagulation can be associated with preventable clinical adverse events [10, 11]. Effective care transitions including patient education, follow-up care and communication have been recommended to reduce the risk of adverse events during care transitions [12, 13]. Despite these recommendations, it is still unclear how institutions with less developed information technology, systems and infrastructure for care transitions can provide patients with this additional layer of safety [10, 13].

Providing patients with sufficient medication education is an essential part of the care plan in order to achieve better patient outcomes [12]. Studies reported improved outcomes when patients were empowered to understand their therapeutic regimens and assume responsibility for the anticoagulation care plan [14].

As compared to other cardiovascular medications, OACs are high-risk medications that require more extensive patient education. Patient education is expected to include information about indications for treatment, benefits, potential side effects, drug intake information, possible food and drug interaction management, alerting signs of bleeding or treatment failure, importance of compliance, and management of missed doses [15]. Such patient education may be even more important with DOACs because of the comparatively shorter half-life of these agents [16].

Recent systematic reviews of patient education for OACs showed no effect on clinical outcomes. These reviews were limited by the low to very low quality of evidence and warranted further research to better assess the effects of supplemental education on clinical outcomes [15, 17].

To our knowledge, there is limited published data evaluating the impact of supplemental education for DOAC use on clinical outcomes such as bleeding.

Limited literature in Lebanon evaluating the impact of the clinical pharmacist and physician counseling on VKA management showed that patients improved their knowledge about factors affecting therapeutic outcomes, and improved medication safety [18].

## Methods

The study was designed to assess the impact of pharmacist-conducted anticoagulation education and follow-up on bleeding and readmission rates. The study design and findings were reported in accordance with the CONSORT 2010 guidelines [19].

This was a randomized, non-blinded interventional study conducted between August 2017 and July 2019 at the Lebanese American University Medical Center – Rizk Hospital (LAUMC-RH), a tertiary care teaching hospital in Beirut, Lebanon. Participants were inpatients aged 18 years and older, admitted to LAUMC-RH, and discharged on an oral anticoagulant for a therapeutic indication. Key exclusion criteria consisted of severe cognitive impairment or altered mental status, unstable psychiatric illness, inability to communicate in Arabic or in English, inability to be followed-up (i.e. does not have a phone, or will be out of reach after discharge), and patients discharged on an anticoagulant for VTE prophylaxis, or otherwise being too ill to participate.

### Patient recruitment and intervention

Eligible patients were identified through the LAUMC-RH Hospital Pharmacy Department, and were approached for written informed consent to participate in the study. One of the study investigators, who was not involved in data collection, performed randomization. Patients were randomized into either the control group or the intervention group by block randomization, with a block size of 4. The control group were patients assigned to receive the standard of care discharge counseling on anticoagulants at the hospital, while the intervention group were assigned to receive a pharmacist-driven discharge counseling on anticoagulants, in addition to the standard of care counseling.

The standard of care discharge counseling at LAUMC-RH was nurse-driven. The process included handing the patients their discharge prescription along with discharge instructions, both of which included the list of discharge medications and instructions. During this process, the nurse would inform the patients about medications to be continued without any modifications, medications for which the dosing regimen had changed, new medications added, and medications that should be stopped. There was no written educational material handed to patients as part of the standard of care. Following the education session, the nurse was expected to document the medication counseling on the “Multidisciplinary Patient/Family Education Form”. The patient and family education form was available in every medical chart, where the nurse would document the learner, the method used, the patient response, and any noted barriers to learning.

Patients who were randomized to the intervention group received a pharmacist-conducted discharge counseling with a focus on their anticoagulant medication, in addition to the regular standard of care. All pharmacists underwent training before they started counseling patients so as to ensure standardization of the information delivered during the counseling session. Every patient was provided with two educational pamphlets. The first pamphlet was common to all anticoagulants and included general information about lifestyle modification while on anticoagulation therapy, management of bleeding if it occurs, general precautions, and information about over-the-counter products that can potentially interact with anticoagulants. The second pamphlet was specific to each anticoagulant and contained information about frequency of dosing, management of missed doses, storage conditions, food and drug interactions, and any information pertinent to the specific anticoagulant. Pharmacists explained the content of both pamphlets to the patient, and to the patient caregiver if warranted, and answered any question related to the drug. A review of patient discharge medications was also provided by the pharmacist. Following the counseling session, the pharmacist documented the education he/she provided in the medical record under two sections: “Multidisciplinary Patient/Family Education Form”, and the “Oral Anticoagulant Pharmacy Education Note”. This hospital-specific form served as a detailed report of the patient education elements discussed during the counseling session.

All patients received two phone calls from the study investigators: one at day 3 and another at day 30 post-discharge to collect relevant patient outcome measures. For the pharmacist-counseled group, at day 3, pharmacists also assessed patients’ knowledge of their anticoagulant medication, clarified any ambiguities, and reached out to their physicians to remediate any identified problem when needed.

### Outcome measures

The primary outcome measures included: 1) readmission rates including unplanned physician’s clinic visit, assessed at day 3 and day 30 post-discharge, 2) any bleeding event (including minor, major, or clinically-relevant non-major bleed, as per the International Society of Thrombosis and Heamostatis – Scientific and Standardization committee (ISTH-SSC) [20, 21], assessed at day 3 and day 30 post-discharge).

The secondary outcome measures included: 1) documented elements of patient education in the medical record and 2) mortality reported during follow-up phone calls at day 30 post-discharge.

### Data collection

The investigators used a data collection form to collect patient demographics, medical history, counseling documentation, concomitant medication use, follow-up calls, bleeding events, and mortality [see Additional File 1]. Upon contacting the patient 2–3 days post-discharge, a Postdischarge Telephone Call Follow-Up Script for Anticoagulation Education adapted from the Agency for Healthcare Research and Quality (AHRQ) was used [see Additional File 2] [22]. Upon contacting the patient 30-days post-discharge, a 30-day Postdischarge Telephone Call Follow-Up Script for Anticoagulation Education was used [see Additional File 3].

### Data management and statistical analysis

Following data collection, the information was coded, entered into SPSS version 24 software, verified for data entry errors, and analyzed. All participants’ responses were reported using descriptive statistics. Means and standard deviations were used to describe continuous variables. Categorical variables were described using frequencies. The association between categorical variables were evaluated using Pearson χ^2^ test or Fisher’s exact test where the expected cell count < 5. Binary logistic regressions were performed to identify factors that affect dichotomous dependent variables (readmission and bleeding outcomes) using Backward LR method. Results were assumed to be significant when *p* < 0.05 for all statistical analysis. All analyses were performed in the intention-to-treat population. No formal power calculation was done.

### Patient and public involvement

Patients or the public were not involved in the design, or conduct, or reporting, or dissemination plans of this research.

## Results

**Fig. 1:**
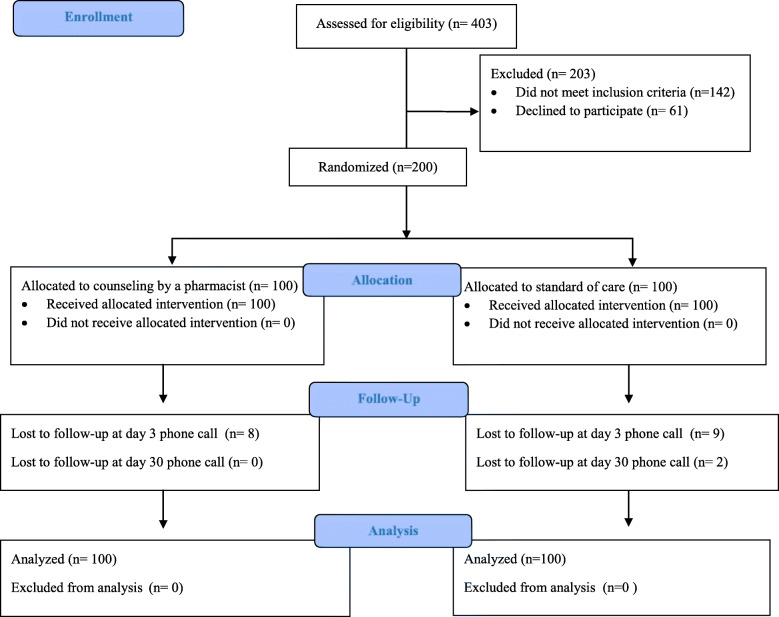
Enrollment Randomization and Follow-Up

A total of 200 patients were included in the study and were randomly assigned to one of the study groups, where 100 patients were counseled solely by a nurse as part of their standard of care, and 100 patients received additional counseling by a pharmacist. Figure 1. Enrollment, Randomization, and Follow-up.

Our patient population included more females than males (55.5% versus 44.5%), and had a mean age of 73.9 years. The most common indication for anticoagulation was atrial fibrillation (73.5%), followed by venous thromboembolism (9.5%), aortic valve replacement (9%), and mitral valve replacement (5.5%). As for baseline characteristics, more patients in the standard of care group received acenocoumarol (50% versus 33%, *p* = 0.015); while more patients in the pharmacist-counseled group received apixaban (15% versus 3%, *p* = 0.005). More patients in the pharmacist-counseled group had a history of heart failure as compared to the standard of care group (34% versus 20%, *p* = 0.026). All other baseline characteristics were similar between the two groups (*p* > 0.05) Table 1.

**Table 1:** Baseline Characteristics

Variable	Pharmacist-counseled ***n*** = 100	Standard of care ***n*** = 100	***P***-value
Sex			0.669
Male	46	43	
Female	54	57	
Age (Mean in years, +/− SD)	74.69 +/− 12.09	73.15+/− 14.74	0.420
Weight (Mean in Kg, +/− SD)	75.52 +/− 18.39	73.60 +/− 17.83	0.465
Smoker	27	26	1.000
Allergic to one or more drugs	22	14	0.141
Creatinine clearance closest to the date of prescribing			0.541
< 15 mL/min	2	1	
15 - < 30 mL/min	5	10	
30 - 50 mL/min	21	19	
> 50 mL/min	51	61	
Insurance type			0.072
Private	37	23	
Public	37	39	
None (self)	26	5	
Indication for anticoagulation			0.236
Atrial fibrillation	79	70	
VTE treatment	8	11	
Aortic valve replacement	7	11	
Mitral valve replacement	4	7	
Antiplatelet use upon admission			0.431
Aspirin 80–100 mg	17	19	
Aspiring 162–325 mg	2	0	
Clopidogrel 75 mg	5	3	
Dual antiplatelet	1	4	
Anticoagulant prescribed upon discharge			0.006
Vitamin K antagonist	33	53	
DOAC	67	47	
DOAC prescribed upon discharge			
Apixaban	15	3	0.005
Dabigatran	12	15	0.535
Rivaroxaban	40	29	0.102
HAS-BLED risk score (Mean +/− SD)	2.26 +/− 1.3	2.23 +/− 1.05	0.882
Past medical history			
Hypertension	71	68	0.645
Heart failure	34	20	0.026
Chronic kidney disease	13	12	0.831
Cancer	13	15	0.684
History of bleeding	8	5	0.390
History of gastrointestinal bleeding	6	4	0.516

*Abbreviations*: *SD* Standard deviation, *VTE* Venous thromboembolism, *DOAC* Direct oral anticoagulant

### Primary outcomes

There was no statistically significant difference in patient readmission rates at day 3 and day 30 post-discharge between the pharmacist-counseled group and the standard of care group. More patients in the pharmacist-counseled group called or had face-to-face contact with a physician within 3 days (14% versus 4%; *p* = 0.010).

Fourteen patients developed bleeding within 30 days post-discharge in the pharmacist-counseled group versus 17 patients in the standard of care group (*p* = 0.700). Within 30 days post-discharge, major bleeding occurred in 2 patients in the pharmacist-counseled group versus 1 patient in the standard of care group (*p* = 0.115). There was no significant difference in the rates of bleeding (minor, major, or clinically relevant) at day 3 and day 30 post-discharge between the two groups. More patients in the pharmacist-counseled group were concomitantly taking an antiplatelet at the time of bleeding (2% versus 1%; *p* = 0.004). The two patients in the pharmacist-counseled group were receiving clopidogrel 75 mg and the patient in the standard of care group was receiving aspirin 81 mg. Bleeding outcomes are detailed in Table 2.

**Table 2:** Primary Outcomes

Variable	Readmission Rates Including Unplanned Physician’s Clinic Visit			
	Pharmacist-counseled ***n*** = 100	Standard of care ***n*** = 100	OR (95% CI)	***P***-value
**Within 3 days**				
Follow-up 1 call completed	92	91		0.621
Patient called or had face-to-face contact with physician (unplanned contact)	14	4	0.238 (0.075–0.754)	0.010
Patient referred to urgent care	2	0		0.369
Patient readmitted (all-cause)	0	0		
**Within 30 days**^a^				
Follow-up 2 call completed	100	97		0.246
Patient had face-to-face contact with physician	13	11	0.928 (0.484–2.453)	0.883
Patient readmitted (all-cause)	15	12	0.847 (0.380–1.886)	0.802
Patient readmitted – related to anticoagulant use	7	7	0.684 (0.193–2.419)	0.650
Time to hospitalization (mean in days, +/− SD)	16.9 +/− 9.8	21.7 +/− 7.1		0.197
**Bleeding Outcomes**				
**Within 3 days**				
Follow-up 1 call completed	92	91		0.621
Bleeding within 3 days	7	4	0.533 (0.151–1.888)	0.483
Minor bleeding	1	2		0.523
Clinically relevant non-major bleeding	5	2		0.333
Major bleeding	1	0		0.435
Bleeding site (within 3 days)				
Nose bleed	1	2		0.693
Gum bleed	0	1		0.593
Blood in sputum	0	1		0.593
Blood in stool	2	1		0.574
Black/dark stool	1	1		0.975
Blood in urine	2	0		0.435
Bruises	1	0		0.435
**Within 30 days**^a^				
Follow-up 2 call completed	100	97		0.246
Bleeding within 30 days	14	17	1.161 (0.544–2.475)	0.700
Apixaban	2	1		
Dabigatran	1	3		
Rivaroxaban	8	6		
Vitamin K antagonist	3	7		
Minor bleeding	4	6	1.532 (0.419–5.603)	0.124
Apixaban	0	1		
Dabigatran	0	1		
Rivaroxaban	3	3		
Vitamin K antagonist	1	1		
Clinically relevant non-major bleeding	8	10	1.263 (0.329–4.848)	0.136
Requiring medical intervention	4	4		0.159
Leading to hospitalization	2	2		0.231
Prompting a face to face evaluation	2	4		0.121
Major bleeding	2	1	0.660 (0.108–4.036)	0.115
Intracranial bleeding	1 (Dabigatran)	0		0.132
Transfusion of more than 2 units of PRBCs	1 (Apixaban)	1 (Rivaroxaban)		0.139
Bleeding site				
Nose bleed	3	2		0.653
Gum bleed	1	2		0.369
Blood in sputum	0	2		0.369
Blood in stool	1	2		0.545
Black/dark stool	1	2		0.708
Blood in urine	2	1		0.291
Blood vomitus	1	0		0.459
Bruises	4	6		0.329

^a^ Reported rates within 30 days are all-inclusive (i.e. include rates within 3 days)

In the multivariable analysis, patients with venous thromboembolism were associated with a higher rate of all-cause readmission within 30 days (*p* = 0.039). There was also a non-statistically significant trend towards heart failure being associated with an increase in all-cause readmission within 30 days (*p* = 0.088). With regards to bleeding, only a history of bleeding was associated with an increase in minor bleeding events within 30 days (*p* = 0.025). Although the HASBLED score and age remained within the final regression model their association with bleeding outcomes was not statistically significant. This could be associated to the relatively small sample size or lack of power. (Table 3 – Regression analysis).

**Table 3:** Regression Analysis

**Readmission Rates Including Unplanned Physician’s Clinic Visit**			
**Patient called or had face-to-face contact with physician within 3 days (unplanned contact)**^**a**^			
**Variable**	**ORa**^**h**^	**95% CI**	**p-Value**
Hypertension	0.211	0.046–0.974	0.046
**Patient readmitted within 30 days (all-cause)**^**b**^			
**Variable**	**ORa**^**h**^	**95% CI**	**p-Value**
Indication of Anticoagulant – VTE Treatment	9.198	1.122–75.389	0.039
Past Medical History – Heart Failure	2.731	0.862–8.562	0.088
**Bleeding Outcomes**			
**Bleeding within 3 days**^**c**^			
**Variable**	**ORa**^**h**^	**95% CI**	**p-Value**
Age	1.068	1.000–1.142	0.051
**Bleeding within 30 days**^**d**^			
**Variable**	**ORa**^**h**^	**95% CI**	**p-Value**
HASBLED Score	1.365	0.959–1.942	0.084
**Minor Bleeding within 30 days**^**e**^			
**Variable**	**ORa**^**h**^	**95% CI**	**p-Value**
History of Bleeding	1.340	1.124–1.602	0.025
**Clinically Relevant Non-major Bleeding within 30 days**^**f**^			
**Variable**	**ORa**^**h**^	**95% CI**	**p-Value**
HASBLED Score	1.416	0.813–2.466	0.219
**Major Bleeding within 30 days**^**g**^			
**Variable**	**ORa**^**h**^	**95% CI**	**p-Value**
History of Bleeding	1.072	0.976–1.162	0.057

^a^ Variables with a p-value of 0.2 or less in the bivariate analysis were included in the initial model. Those include: Age, Allergic to one or more drug, Hypertension, CKD, and history of GI Bleeding. Using Backward LR method, the model finally retained the variables shown in this table. Hosmer and Lemshow test for sample adequacy *p*-value: 0.907

^b^Variables with a p-value of 0.2 or less in the bivariate analysis were included in the initial model. Those include: Smoker, Indication of anticoagulant, the anticoagulant prescribed, and Heart Failure as a past medical history.  Using ENTER method, the model finally retained the variables shown in this table. Hosmer and Lemshow test for sample adequacy p-value: 0.952.

^c^ Variables with a p-value of 0.2 or less in the bivariate analysis were included in the initial model. Those include: Age, Weight, Smoker, and Indication of anticoagulants. Using Backward LR method, the model finally retained the variables shown in this table. Hosmer and Lemshow test for sample adequacy *p*-value: 0.404

^d^ Variables with a p-value of 0.2 or less in the bivariate analysis were included in the initial model. Those include:Age, History of Bleeding, Hypertension as a medical history, Indication for anticoagulants, and HASBLED score. Using Backward LR method, the model finally retained the variables shown in this table. Hosmer and Lemshow test for sample adequacy *p*-value: 0.647.

e Variables with a p-value of 0.2 or less in the bivariate analysis were included in the initial model. Those include: BMI, Gender, Indication of anticoagulant, and history of bleeding. Using Backward LR method, the model finally retained the variables shown in this table. Hosmer and Lemshow test for sample adequacy *p*-value: 0.435.

f Variables with a p-value of 0.2 or less in the bivariate analysis were included in the initial model. Those include: Allergic to one or more drug, Antiplatelet use upon admission, Cancer, and HASBLED score. Using Backward LR method, the model finally retained the variables shown in this table. Hosmer and Lemshow test for sample adequacy p-value: 0.408.

g Variables with a *p*-value of 0.2 or less in the bivariate analysis were included in the initial model. Those include: History of Allergy, Hypertension as a medical history, History of bleeding, and HASBLED score of more than 3. Using Backward LR method, the model finally retained the variables shown in this table. Hosmer and Lemshow test for sample adequacy *p*-value: 0.721.

^h^ORa: Odds ratio (adjusted)

### Secondary outcomes

Pharmacists had significantly better documentation of the counseling session including the method used, the patient response, and any identified patient barrier to learning (*p* < 0.05). In addition, pharmacists provided a more explicit documentation of all elements of patient education, including rationale for therapy, dosing and administration, monitoring, duration of therapy, patient communication with healthcare provider, etc. (*p* < 0.001). Among all elements of counseling, pharmacists documented counseling the least about reversal agents (only 32%). More patients in the standard of care group were informed about next appointment date with physician, as compared to the pharmacist-counseled group (52% versus 31%, *p* < 0.001). Details about counseling documentation are stated in Table 4.

**Table 4:** Counseling Documentation

Variable	Pharmacist counseled ***n*** = 100	Standard of care ***n*** = 100	***P***-value
Learner specified^a^	97	34	< 0.001
Patient	82	34	< 0.001
Family member	77	20	< 0.001
Other	1	0	0.014
Counseling method specified^b^	97	33	< 0.001
Verbal education	97	33	< 0.001
Written educational material	95	0	< 0.001
Language of written education material			
Arabic	18	2	< 0.001
English	40	10	< 0.001
Not documented	42	88	< 0.001
Response of patient documented	100	33	< 0.001
Patient verbalized understanding	100	33	< 0.001
Patient needs reinforcement; re-evaluate education at another time	2	1	0.014
Instructions declined	1	0	0.014
Documented barriers to learning	65	33	< 0.001
Age-related	6	0	< 0.001
Cognitive	2	0	0.007
Emotional	1	0	0.014
Hearing	1	0	0.014
No Barrier	84	33	< 0.001
Elements of patient education			
Rationale for therapy	92	5	< 0.001
Dosing	91	5	< 0.001
Monitoring	91	5	< 0.001
INR (for patients receiving Vitamin K antagonist)	33	2	< 0.001
Duration of therapy	82	5	< 0.001
Administration	92	5	< 0.001
Importance of compliance	94	5	< 0.001
Missed dose	91	5	< 0.001
Storage	88	5	< 0.001
Drug-drug interaction	88	5	< 0.001
Drug-food interaction	88	5	< 0.001
Signs and symptoms of bleeding	90	5	< 0.001
Reversal agent	32	2	< 0.001
Precautions	93	5	< 0.001
Patient communication with healthcare provider	41	1	< 0.001
Discharge medications documented in chart	94	84	0.024
Informed patient about next appointment date with physician	31	52	< 0.001
Time of next appointment (mean in days, +/− SD)	15.00 +/− 12.49	17.67 +/− 14.53	0.341

^a^ The values add up to more than 100% since more than one learner can exist for each counseling session (i.e. patient and family)

^b^ The values add up to more than 100% since more than one counseling method can be used in each counseling session (i.e. verbal education and written education material)

There was no significant difference in patient mortality at 30 days post-discharge (2 patients in the pharmacist-counseled group versus 4 patients in the standard of care group; *p* = 0.724). Table 5**.**

**Table 5:** Mortality (30 days)

Variable	Pharmacist- counseled ***n*** = 100	Standard of care ***n*** = 100	***P***-value
Follow-up 2 call completed	100	97	0.246
Mortality	2	4	0.724
Cardiac arrest	0	2	
Cancer	1	1	
Heart failure	0	1	
COPD	1	0	
Mortality day number after prescription (Mean in days, +/− SD)	1.3 +/− 4.6	6.6 +/− 10.5	0.326

### Other outcomes

At day 3 post-discharge, 87% of the patients in the pharmacist-counseled group knew the name of their medication, 91% knew the correct frequency, and 90% knew the correct strength, dose and special instructions. Only 55% of these patients knew the reason for taking their medication. All missing information related to anticoagulation was clarified for the patients by the study investigators during follow-up. All patients receiving vitamin K antagonists were aware of the schedule of their next international normalized ratio (INR) test, and knew who to call for their INR results.

Fourteen patients in the pharmacist-counseled group reported having problems with their medications, including a transportation barrier to perform INR test (2 patients), financial barrier to acquire the anticoagulant medication (5 patients), and side effects problems (7 patients). The pharmacist reached out to the treating physician in 2 cases to remediate identified issues specifically pertaining to incorrect dosing frequency of dabigatran and the inability of the patient to start their medication due to lack of availability at the outpatient community pharmacy. In both situations, the physicians followed-up with the patients to rectify the problems.

## Discussion

In this randomized clinical trial, pharmacist-conducted anticoagulation education did not appear to reduce bleeding or readmission rates at 30 days. However, pharmacist education and post-discharge follow-up on anticoagulation therapy significantly increased patient communication with their providers within 3 days post-discharge.

The impact of pharmacist-led discharge counseling on hospital readmission and emergency department visit has been well studied in different patient populations and clinical conditions [23–26].

Similar to our results, many of these studies were not able to demonstrate significant differences between usual care and pharmacist interventions in the transition of care. Few studies assessing the impact of pharmacist-conducted anticoagulant education programs showed significant reductions in readmission rates [27, 28]. This difference in findings could be attributed to many reasons including heterogeneity and complexity of pharmacist interventions, different study design (interventional versus retrospective review) and the small sample size. In our study, during telephone follow-up on day 3, the investigators had to speak with the patient’s caregiver when they could not reach the patient. This could have impacted the patient’s understanding about follow-up care and may have created patient education breakdowns. In fact, the Joint Commission describes the following root causes of ineffective transitions of care: communication breakdowns, patient education breakdowns, and accountability breakdowns [8]. Moreover, the 2020 NPSG.03.05.01 and NPSG.03.06.01 highlight the importance of documentation of the initiation and maintenance of anticoagulant therapy [29]. While pharmacists performed a thorough patient education covering all the elements of anticoagulation counseling, they did not consistently close the loop and document the discharge instructions in the patients’ medical charts as compared to healthcare providers performing the standard of care. This constitutes a quality improvement area that warrants the consideration of the Pharmacy Department. In contrast, nurses informed patients and documented the date of their next appointment in the charts. Lastly, our intervention did not include a formal medication reconciliation for all patients prior to discharge. These reasons could have contributed to the findings.

Although pharmacist intervention did not reduce readmission rates in our study, patients counseled by pharmacists established a better communication with healthcare providers, as evidenced by significantly more clinic visits and calls within 3 days. We believe this can be explained as patients became more cognizant of their anticoagulant side effect and/or need for follow-up. In the binary logistic regression, patients with VTE were associated with a higher rate of all-cause readmission within 30 days whereas CHF showed a trend towards higher readmission rates. This finding is in congruence with published literature showing acute VTE patients associated with a high burden of 30-day readmissions [30]. Similarly CHF as one of the most common cause of readmissions [31].

The study found no difference in the bleeding outcomes between the pharmacist-counseled group and the standard-of-care group. The fact that we included patients on all oral anticoagulants and for different indications, and had a relatively small sample size may have limited the ability to discern any differences between the groups. In fact, VKAs and the different DOACs have shown different bleeding profiles in different patient populations [32–34]. It is worth noting that the majority of the bleeding events in our study were observed in patients prescribed DOACs within each arm (11 out of 14 in the pharmacist counseled arm and 10 out of 17 in the standard of care arm) but this study was not designed to compare the bleeding rates between VKA and DOACs. Moreover, in the multivariate logistic regression, a history of bleeding was associated with the occurrence of minor bleeding events within 30 days. Although not statistically significant, there was a trend towards a significant association between the HASBLED score and major bleeding within 30 days, consistent with published literature [35]. This may have been due to the fact that our patient population comprised AFib and VTE, in which the HASBLED score has shown good predictive validity but has not been as extensively studied [36].

### Study limitations

We acknowledge the potential weaknesses and limitations of this study that might limit the generalizability of the results. The single-centered study design, the lack of power calculation and the small sample size may have decreased the possibility of detecting statistically significant difference in bleeding rates between groups. Due to the nature of the intervention, this was an open-label, non-blinded study which could have biased the results. However the findings of the study suggest that this limitation is unlikely to have had an influence on the study effect. Pharmacist investigators were only available during weekdays. To avoid bias, patients randomized to the intervention group who were planned for discharge during the weekend, received pharmacist-counseling before the weekend.

## Conclusion

Although pharmacist-conducted anticoagulation education did not appear to reduce bleeding or readmission rates at 30 days, pharmacist education significantly increased patient communication with their providers in the early days post-discharge.

## Supplementary Information

**Additional file 1.** Case report form. Data collection form to collect patient demographics, medical history, counseling documentation, concomitant medication use, follow-up calls, bleeding events, and mortality.

**Additional file 2.** 48–72 Hours Post Discharge Telephone Follow-up Script for Pharmacist-Driven Anticoagulation Education. Script used at day 3 to assess patients’ knowledge of their anticoagulant medication, clarify any ambiguities, identify any problem when needed, and assess bleeding and readmission outcomes.

**Additional file 3.** Script for follow-up telephone call: 30 days post education. Script used at day 30 to assess readmissions, contact with health care providers or any bleeding event.

## Data Availability

The datasets used and/or analyzed during the current study are available from the corresponding author on reasonable request.

## Abbreviations

ADEs: Adverse Drug Events
AHRQ: Agency for Healthcare Research and Quality
DOACs: Direct oral anticoagulants
INR: International normalized ratio
IRB: Institutional Review Board
ISTH-SSC: International Society of Thrombosis and Heamostatis – Scientific and Standardization committee
LAUMC-RH: Lebanese American University Medical Center – Rizk Hospital
LBCTR: Lebanese Clinical Trials Registry
NPSG: National Patient Safety Goal
OACs: Oral anticoagulants (OACs)
VKAs: Vitamin K antagonists
VTE: Venous thromboembolism
