# Supplementary material for: Impact of pharmacist-conducted anticoagulation patient education and telephone follow-up on transitions of care: a randomized controlled trial
Source: BMC Health Serv Res. 2021 Feb 16;21:151. doi: 10.1186/s12913-021-06156-2 (PMC7885504; doi:10.1186/s12913-021-06156-2)
Supplement: Supplementary file 1 — Additional file 1. Case report form. Data collection form to collect patient demographics, medical history, counseling documentation, concomitant medication use, follow-up calls, bleeding events, and mortality. [file 12913_2021_6156_MOESM1_ESM.pdf]

Appendix 1

CASE REPORT FORM

Impact of pharmacist-conducted anticoagulation patient education and telephone follow-up on transitions of care: a randomized controlled trial

Patient Initials ☐☐☐ **Subject No.** ☐☐☐

FOLLOW-UP TELEPHONE CONTACT 40

Contacts

Name of Contact 1: ______________________________

Relationship: ______________________________

Caregiver? (Y/N) _____

Proxy? (Y/N) ______

Designated to receive followup phone call? (Y/N) ______

Notes: ______________________________

____

Preferred Spoken Language

____

Interpreter needed? (Y / N)

Preferred phone number: ___ home ___ cell phone ____work

Home Phone: (_____) ____________________ OK to leave message? (Y/N) ______

Best time to call: ____________________

Cell Phone: (_____) ____________________ OK to leave message? (Y/N) ______

Best time to call: ____________________

Work Phone: (_____) ____________________ OK to leave message? (Y/N) ______

Best time to call: ____________________

PART I: Patient Demographics

Patient Identification Number (MRN)	Year of Birth	Gender<br>☐ M ☐ F	Smoker<br>☐ NO<br>☐ YES	Weight (Kg): ________<br>Height (cm): ________<br>BMI (Kg/ m^2): _______	Insurance type<br>☐ Private ☐ Public ☐ None	
Allergies<br>☐ NO ☐ YES ________________		Date of Admission: **dd/mm/yr** ____________	Chief complaint	Admission Diagnosis	Date of Discharge: **dd/mm/yr**________	Hospital LOS(d):_____

Inclusion Criteria:
- ☐ Age > 18 years, discharged on any therapeutic anticoagulant including vitamin K antiagonists, non-vitamin K antagonist oral anticoagulants (apixaban, rivaroxaban, dabigatran), or parenteral anticoagulant for reasons other than venous thromboembolism prophylaxis.
- ☐ **Discharge anticoagulation counseling documented on patient and family education form in the medical record**
- ☐ For whom communication via telephone calls was possible for follow up. Patients who had recently undergone surgery were also included if they were on therapeutic anticoagulation for a non-surgical indication.

Exclusion Criteria:
- ☐ Patients discharged on anticoagulants for thromboembolism prophylaxis
- ☐ Orthopedic thromboprophylaxis
- ☐ Prescribed treatment duration less than 1 month

Indication for Anticoagulant
- ☐ Atrial Fibrillation
- ☐ VTE treatment
- ☐ Other ____________

Prescribing Service
- ☐ Cardiology
- ☐ Family Medicine
- ☐ Haematology/Oncology
- ☐ Infectious Diseases
- ☐ Internal Medicine
- ☐ Neurology
- ☐ Orthopaedic Surgery
- ☐ Plastic Surgery
- ☐ Pulmonary
- ☐ Vascular Surgery
- ☐ Other__________

Antiplatelet use on admission
☐ NO ☐ YES

Specify antiplatelet therapy
- ☐ aspirin 80-100mg
- ☐ aspirin > 162-325mg
- ☐ clopidogrel 75mg
- ☐ prasugrel 10mg
- ☐ ticagrelor 90mg
- ☐ dual antiplatelet

Creatinine clearance closest to the date that the discharge anticoagulant was prescribed _________ ml/min

Anticoagulant prescribed on discharge
- ☐ Acenocoumarol (sintrom)
- ☐ Enoxaparin
- ☐ Tinzaparin
- ☐ Fondaparinux
- ☐ Apixaban
- ☐ Rivaroxaban
- ☐ Dabigatran
- ☐ Fluindinedione
- ☐ Hydroxycoumarol (warfarin)
- ☐ Other

Anticoagulant Dose ______________ (mg or units)

Frequency
- ☐ Once daily
- ☐ Bid
- ☐ Q 48 hours
- ☐ Other __________

**PART II Medical History**	
**PART II.i Bleeding Risk in Anticoagulated Patients with Atrial Fibrillation based on the HAS-BLED RISK SCORE: TOTAL SCORE ____________________ (In the final score, only add up the major points)**	
**1 point/ risk factor**	
☐ Hypertension<br>☐ Abnormal renal function<br>☐ (chronic dialysis<br>☐ renal transplant<br>☐ serum creatinine ≥ 2.3 mg/dL (200 µmol/L)<br>☐ Abnormal liver function<br>☐ (cirrhosis<br>☐ bilirubin > 2x UNL with AST/ALT/AP > 3x UNL)<br>☐ Stroke	☐ Bleeding history<br>☐ or predisposition (anemia)<br>☐ Labile INR (Therapeutic time in range < 60%)<br>☐ Elderly (greater than 65 years old)<br>☐ Drugs<br>☐ (other antiplatelet agents<br>☐ or NSAIDs)<br>☐ Alcohol (more than 8 drinks per week)

Medical Conditions:		
☐HTN ☐Dyslipidemia ☐CHF ☐Asthma ☐COPD ☐GERD ☐DVT ☐Arthritis<br>☐Seizure ☐CKD ☐Depression ☐Constipation ☐Insomnia ☐Parkinson ☐Alzheimer<br>☐Cancer ☐DM ☐ History of bleeding ☐Other_______<br>☐ Age> 65 years<br>☐ history of gastrointestinal bleeding<br>☐ history of stroke,and at least one of the following variables: myocardial infarction, hematocrit< 30%, creatinine >1.5 mg/dL, and diabetes.<br>☐ prior bleeding	☐ anemia<br>☐ genetic factors(CYP2C9 polymorphism)<br>☐ an excessive risk of falls<br>☐ Recent surgery on critical organ (brain; eye)<br>☐ Thrombocytopenia (e.g. chemotherapy)	☐ HAS-BLED ≥3<br>☐ ≥65 years<br>☐ ≥75 years<br>☐ ≥80 years<br>☐ Weight<60 (Kg)<br>☐ reduced platelet count or function<br>☐ hypertension (uncontrolled)

☐ hepatic or renal disease<br>☐ ethanol abuse<br>☐ malignancy<br>☐ age>75 years		☐ Previous Surgical History: ________________

PART III: Counseling Documentation

REFER TO THE MULTIDISCIPLINARY PATIENT & FAMILY NOTE

Discharge anticoagulation counseling performed by:

Student pharmacist ☐ NO ☐ YES
Faculty ☐ NO ☐ YES
Full time clinical pharmacist ☐ NO ☐ YES
Hospital pharmacist ☐ NO ☐ YES
Nurse ☐ NO ☐ YES
Physician ☐ NO ☐ YES

Were the following criteria filled on the patient and family education form

Was the Learner specified: ☐ NO ☐ YES
Patient ☐ NO ☐ YES
Family ☐ NO ☐ YES
Other ☐ NO ☐ YES

Was the counseling Method specified : ☐ NO ☐ YES
Verbal ☐ NO ☐ YES
Instructions Sheet ☐ NO ☐ YES

Language of Written material Education:
English ☐ NO ☐ YES
Arabic ☐ NO ☐ YES
Not documented ☐ NO ☐ YES

Was the Response of Patient documented: ☐ NO ☐ YES

Verbalized understanding☐ NO ☐ YES

Needs reinforcement; re-evaluate education at another time ☐ NO ☐ YES

Instructions declined ☐ NO ☐ YES

Did the educator document in the section of Barriers to Learning: ☐ NO ☐ YES

Was the specific anticoagulation counseling documented <u>on oral anticoagulation documentation form:</u> ☐NO ☐YES

Were the specific patient Learning Needs identified☐NO ☐ YES
Rationale for therapy ☐ NO ☐ YES
Dosing ☐ NO ☐ YES
Monitoring ☐ NO ☐ YES
INR ☐ NO ☐ YES
Duration of therapy ☐ NO ☐ YES
Administration ☐ NO ☐ YES

Importance of compliance ☐ NO ☐ YES
How to handle a missed dose ☐ NO ☐ YES
Storage ☐ NO ☐ YES
Drug-Drug interaction ☐ NO ☐ YES
Drug-Food interactions ☐ NO ☐ YES

Common side effects ☐ NO ☐ YES
Signs/Symptoms of Bleeding and Clotting ☐ NO ☐ YES
Reversal agent ☐ NO ☐ YES
General precautions / patient safety issues ☐ NO ☐ YES

Communication with healthcare provider (follow-up, planned surgeries, etc) ☐ NO ☐ YES

NOTES: ____
____

Recommendations:
____
____

<u>Refer to the Hospital Discharge Summary of Unified Prescription</u>:

Were the discharge medications available? ☐ NO ☐YES

If yes, Discharge medications available on (select all that apply):
☐ Discharge summary ☐Unified prescription ☐Other______________

Pre-specified time period for next appointment or contact with physician is specified (Health care professional communicated this information with the patient ☐ NO ☐YES, **specify date and calculate days post discharge________________**

Emergency contact number provided to patients in case of any issues with barriers in care (ex. physician number or hospital number)
☐ NO ☐ YES

<u>Other Information (May be found in the Multidisciplinary or Anticoagulation education note)</u>
Verbally communicated any ambiguities with the primary physician that arose during counseling the patient. ☐ NO ☐ YES

Demonstration of patient ability and comfort to self-administer parenteral anticoagulant ☐ NO ☐ YES

Assurance of affordability, insurance coverage and retail availability of anticoagulant therapies

Age Related ☐ NO ☐ YES<br>Cognitive ☐ NO ☐ YES<br>Cultural ☐ NO ☐ YES<br>Emotional ☐ NO ☐ YES<br>Hearing ☐ NO ☐ YES<br>Language ☐ NO ☐ YES<br>Reading ☐ NO ☐ YES<br>Religious ☐ NO ☐ YES<br>Visual ☐ NO ☐ YES<br>No Barrier ☐ NO ☐ YES	**How much time did the pharmacist allocate to counseling the patient? ________________________________**	☐ NO ☐ YES<br><br>Appropriate and accurate prescriptions given to patient prior to discharge ☐ NO ☐ YES<br><br>Inpatient dosing history, discharge instructions and discharge summary sent to receiving provider in time to allow for receiving provider to effectively care for the patient ☐ NO ☐ YES

PART IV: Discharge medications and concomitant medications on the discharge instructions sheet or the Unified Prescription:

Medications possibly affecting Anticoagulant dose:

☐ Concomitant use of P-gp inhibitor
☐ Strong CYP3A4 inhibitor
☐ Verapamil containing medicines
☐ Diltiazem
☐ Quinidine
☐ Amiodarone
☐ Dronedarone
☐ Clarithromycin
☐ Erythromycin
☐ Carbamazepine
☐ Phenobarbital
☐ Phenytoin
☐ St John's wort
☐ HIV protease inhibitors (e.g. ritonavir)
☐ Rifampicin
☐ Cyclosporin
☐ Tacrolimus
☐ Itraconazole
☐ Ketoconazole
☐ Posaconazole
☐ Voriconazole
☐ Fluconazole
☐ Naproxen

Antiplatelet use on discharge

☐ NO ☐ YES

Specify antiplatelet therapy

Medications possibly interacting with Vitamin K antagonist

☐ Ciprofloxacin
☐ Amiodarone
☐ Phenylbutazone
☐ Alcohol (if concomitant liver disease)
☐ Cimetidine
☐ Boldo-fenugreek
☐ Anabolic steroids
☐ Cotrimoxazole
☐ Clofibrate
☐ Piroxicam
☐ Fish oil
☐ Quilinggao
☐ Zileuton
☐ Erythromycin
☐ Diltiazem
☐ Citalopram
☐ Mango
☐ Entacapone
☐ Omeprazole
☐ Fluconazole
☐ Fenofi brate
☐ Sertraline
☐ Isoniazid
☐ Propafenone
☐ Metronidazole
☐ Propranolol

LIST THE DISCHARGE MEDICATIONS (Available from Medications on discharge instructions sheet or the Unified Drug Prescription:

1. .
2. .
3. .
4. .
5. .
6.
7. .
8. .
9.
10. .
11.
12. .
13.
14.

☐ aspirin 80-100mg<br>☐ aspirin > 162-325mg<br>☐ clopidogrel 75mg<br>☐ prasugrel 10mg<br>☐ ticagrelor 90mg<br>☐ dual antiplatelet____________<br><br>☐ **NSAIDS**<br>☐ **STEROIDS**	☐ Miconazole oral gel<br>☐ Miconazole intravaginal<br>☐ Sulfinpyrazone<br>☐ Griseofulvin<br>☐ Cholestyramine<br>☐ Mesalamine<br>☐ Barbiturates<br>☐ High vitamin K content foods/enteral feeds<br>☐ Mercaptopurine<br>☐ Nafcillin<br>☐ Carbamazepine<br>☐ Avocado (large amounts)<br>☐ Ribavirin Rifampin	15. .<br>16. .<br>17. .

**Part V: Follow up 1** ***(Use the telephone script to document findings. If patient is readmitted & you have access to their chart, use the chart for follow up and document bleeding findings into the table below)***	
Follow up phone call within 48-72 hours<br>☐ NO ☐ YES	If Bleeding, **YES DURING FOLLOW UP**. **Fill in description of bleeding below.**

**Part VI: Follow up 2:** ***(Use the telephone script to document findings. If patient is readmitted & you have access to their chart, use the chart for follow up and document bleeding findings into the table below)***	
Follow up phone call on day 30<br>☐ NO ☐ YES	If Bleeding, **YES DURING FOLLOW UP**. **Fill in description of bleeding below.**

Interpret the Bleeding Findings(From the follow up 1&2) according to the definitions below and fill in the table accordingly
Bleeding Event (BE) as per International Society of Thrombosis and Haemostasis and SCC

**Major bleeding**		**Clinically relevant non-major bleed**	**Minor bleed**	
☐ Intracranial<br>☐ Retroperitoneal<br>☐ Intraocular<br>☐ Intra-articular<br>☐ Pericardial	☐ Hg drop ≥2 mg/dl<br>☐ Fatal Bleed<br>☐ Transfusion > 2 units of PRBCs<br>☐ Spinal<br>☐ Intramuscular with	☐ Requiring medical intervention by a healthcare professional<br>☐ Leading to hospitalization or increased level of care<br>☐ Prompting a face to face evaluation	☐ Sign or symptom of hemorrhage that doesn't fit the definitions of major bleed and clinically relevant non-major bleed requiring just telephone or electronic communication without	☐ BE on day# after discharge prescription: _______<br>☐ BE while on concomitant antiplatelet or on an anticoagulant other than the anticoagulant discharged on: yes or no<br><br>☐ Document the type of bleed according to the

	compartment syndrome		needing to see the physician	follow up 1 and 2:<br>Follow up #1:_________<br>Follow up #2:_______________

Mortality ☐ yes ☐ No Reason for mortality: ___________________________________
☐ Mortality on day# after prescription: ___________

Date	INR	PT	PTT	Platelets	Hct	Hb	SrCr	CrCl
Admission date____								

**Anticoagulation medications administered**				
Date /time	Agent	Dose	Route	Frequency
☐ Total number of doses received during inpatient stay________________				
