# Supplementary material for: Impact of pharmacist-conducted anticoagulation patient education and telephone follow-up on transitions of care: a randomized controlled trial
Source: BMC Health Serv Res. 2021 Feb 16;21:151. doi: 10.1186/s12913-021-06156-2 (PMC7885504; doi:10.1186/s12913-021-06156-2)
Supplement: Supplementary file 2 — Additional file 2. 48–72 Hours Post Discharge Telephone Follow-up Script for Pharmacist-Driven Anticoagulation Education. Script used at day 3 to assess patients’ knowledge of their anticoagulant medication, clarify any ambiguities, identify any problem when needed, and assess bleeding and readmission outcomes. [file 12913_2021_6156_MOESM2_ESM.pdf]

Appendix 2.

48-72 Hours Post Discharge Telephone Follow-up Script for Pharmacist-Driven Anticoagulation Education

Prior to phone call **Review and make sure to have the following information INFRONT OF YOU:**

- ☐ Health history
- ☐ Discharge medication list
- ☐ Contact sheet
- ☐ Educator notes on the Patient and Family education note or on the Oral anticoagulant education note.
- ☐ Discharge summary

Call completed ❑ Yes ❑ No

- Specify if call was on day 2 or 3 post discharge ❑ day2 ❑ day3
- With whom (patient, caregiver, both): ___________________________

Consultations (if any) made prior to phone call:

- ❑ None
- ❑ Called MD
- ❑ Called Discharge educator
- ❑ Other: ____

If any consultations, note to whom you spoke, regarding what, and with what outcome:

____

____

Phone Call Attempts

Patient/Proxy

Phone Call #1: Date & Time:________ Reached: Yes/No

Phone Call #1: Date & Time:________ Reached: Yes/No

If No (circle one): ans. machine/no answer/not home/declined to provide information/busy/other:

Phone Call #2: Date & Time:________ Reached: Yes/No

If No (circle one): ans. machine/no answer/not home/declined to provide information/busy/other:

Phone Call #3: Date & Time:________ Reached: Yes/No

If No (circle one): ans. machine/no answer/not home/declined to provide information/busy/other:

Phone Call #4: Date & Time:________ Reached: Yes/No

Alternate Contact 1

Alternate Contact 2

Phone Call #1: Date & Time:________ Reached: Yes/No

If No (circle one): ans. machine/no answer/not home/declined to provide information/busy/other:

Phone Call #2: Date & Time:________ Reached: Yes/No

If No (circle one): ans. machine/no answer/not home/declined to provide information/busy/other:

Phone Call #3: Date & Time:________ Reached: Yes/No

If No (circle one): ans. machine/no answer/not home/declined to provide information/busy/other:

Phone Call #4: Date & Time:________ Reached: Yes/No

Opening Statement

Hello, can I please speak with ______________________ (name of study subject or whomever signed the consent form). This is Dr. __________________ calling you as a follow up to the study that you approved to be involved in during your stay at LAUMCRH. As you know, we shared with you that we would call you between 2-3 days after leaving the hospital to ask you if you had any questions or concerns about your blood thinner. I just wanted to ask you a few questions. Since you left the hospital, I am hoping to talk to you about your medications. Do you mind if I ask you a few questions? Is this a good time to talk? It will probably take about 10 to 15 minutes, depending on the number of medicines you are taking.

If yes, continue.
If no, CALLER: Is there a better time that I can call you back?

A. Diagnosis and Health Status

CALLER: Before you left the hospital, [Discharge Educator_______________________(state name if available on data collection sheet, or the profession if the name is illegible] spoke to you about your use of blood thinners for your (Anticoagulant indication) during your hospital stay. Using your own words, can you explain why you are on the blood thinner?

- **If yes,** confirm the patient's knowledge of indication for anticoagulation by using the "teach-back" method. After the patient describes his or her indication for anticoagulation, clarify any misconceptions or misunderstandings using a question and answer format to keep the patient engaged. □Patient confirmed understanding

- **If no,** use this opportunity to provide patient education about the anticoagulant indication. Then conduct teach-back to confirm the patient understood. □Further instruction was needed

B. Medications

CALLER: I will specifically ask you questions about your blood thinner now. Can you bring all of your medicines to the phone, please? We will review them during this call. Bring both prescription medicines and over-the-counter medicines, the ones you can buy at pharmacy without a doctor's prescription. Also, bring any supplements such as herbs, you are taking. Do you still have the list or unified prescription that we gave you before you left the hospital?

CALLER: Do you have all of your medicines in front of you now?

CALLER: I'm going to ask you a few questions about the list of your medications and specifically the blood thinner medications.. We will go through your medicines one by one.

Choose the anticoagulant to start with.

CALLER:

- What is the name of this medicine? Knows the name of the medicine? ❑ Yes ❑ No

- At what times during the day do you take this medicine? Takes correct frequency in accordance with the discharge prescription ❑ Yes ❑ No

- How much do you take each time? Takes correct dose in accordance with the discharge prescription❑ Yes ❑ No (note: **If the patient answers in terms of how many pills, lozenges, suppositories, etc.)**

- What is the strength of the medicine? It should say a number and a unit such as mg or mcg. **If the patient answers in terms of how many pills, lozenges, suppositories, etc.** Takes correct strength of the medicine in accordance to the prescription? ❑ Yes ❑ NO

- How do you take this medicine? **If there are special instructions** (e.g., take with food), probe as to whether the patient knows the instructions and whether he or she is taking the medicine as instructed. Takes medication correctly i**f there are special instructions** (e.g., take with food) ❑ Yes ❑ No

- Why do you take this medication? (example AFIB, STROKE)
  - ☐ Afib
  - ☐ Stroke
  - ☐ PE
  - ☐ DVT
  - ☐ Just said anticoagulant- doesn't know

- Have you had any concerns or problems taking this medicine? Has anything gotten in the way of your being able to take it? Have you ever missed taking this medicine when you were supposed to? Why?
  Expresses any concerns or problems taking this medicine? ❑ Yes ❑ No
  Specify:________________________________

- Do you think you are experiencing any side effects from the medicine?**If yes,** Could you please describe these side effects? Describe side effects.____________________________

CALLER: Have you experienced any bleeding within these 3 days? ❑ Yes ❑ No

If no, acknowledge that it's a good thing and continue taking it as is.

CALLER:

- **Did you have any of these problems?**

	Yes	No
Blood from your nose	☐	☐
Blood from your gum	☐	☐
Blood or redness in your eyes	☐	☐
Blood in the sputum	☐	☐
Purple /greenish bruises on your body	☐	☐
Blood in vomitus	☐	☐
Blood in stools	☐	☐
Dark black stools	☐	☐
Blood in urine	☐	☐
Any major bleeding such as in head, stomach, did you have to receive blood?	☐	☐

- Did you have to call or see your doctor? ❑ Yes ❑ No
  If yes, specify

  - ☐ Contacted or seen clinician? (name): ___________________________________
  - ☐ Gone to the ER/urgent care? (specify):___________________________________
  - ☐ Gone to another hospital/MD? (name): _________________________________
  - ☐ Other

- **CALLER:** I'm going to ask you about your list of your medications. Can you tell me what medications you are taking now***? (Don't mention that you have the list that was***

given upon discharge. If there are any discrepancies, we will notify the MD and then maybe call the patient back after communicating with the physician).

- **LIST THE MEDICATIONS:**

- **Current medication list consistent with discharge medication list?**
  - ☐ **Yes**
  - ☐ **No, Medication that a patient is taking and it's NOT on discharge summary or unified prescription**
  - ☐ **No, Medication that patient is NOT TAKING, although it was on the discharge summary or unified prescription (e.g., has not obtained, is not taking correctly, has concerns, including side effects)**
  - ☐ **Discharge medication list was not available to start with**

<u>MISSING MEDICATION:</u>

- Name of medication(s)missing ____

If patient has been prescribed medications (according to the discharge summary or unified prescription) that the patient has not mentioned, ask the patient casually without making it sound like he/she is missing something, whether he or she is taking that medicine.

- **CALLER: Are you taking (______________________) ? (*The missing medication)***
  - ☐ **Yes, patient confirmed. Just add to the list of medications above.**
  - ☐ **No, ➔ ASK PATIENT**
- **CALLER: Oh, okay, so you're <u>not</u> taking (______________________) did you stop it or never started it?**

<u>Make a note to check with discharge physician as to whether patient is supposed to be taking it.</u> Document any medication discrepancies below and NOTIFY PRINCIPAL INVESTIGATOR IMMEDIATELY.

- Specify if the Problem was:

❑ Intentional nonadherence
❑ Inadvertent nonadherence
❑ System/provider error

NEW MEDICATION:

Name of medication(s) that were newly started: ____

- **CALLER: Is (_______________________) a new medication that you've started? Who prescribed it for you? _________________, When was it started?_____________**
  - ☐ **Yes, it's new and my doctor is aware**
  - ☐ **Yes, it's new and I haven't told my doctor yet.**
  - ☐ **No, it's a chronic medication that I've been on**

- **Make a note to notify the discharge physician that the patient was newly started on:**
- **Name of medication(s): _____________________**
- **medication(s) started on date:_____________________________**
- **Prescribed by provider:____________________________________**

- Specify if the Problem was:

❑ Intentional nonadherence
❑ Inadvertent nonadherence
❑ System/provider error

- Any other problem based on the questions asked? ❑ Yes ❑ No

Name of medication (s) ____

Specify if the Problem was:

❑ Intentional nonadherence
❑ Inadvertent nonadherence
❑ System/provider error

- **CALLER:** Do you use a pill box?

  If yes, provide positive reinforcement of using this tool.
  If no, suggest using this tool to help remember to take the medicines?
  - ☐ **YES**
  - ☐ NO

C. Clarification of INR and Appointments

- **CALLER:** Now, I'm going to make sure you and I have the same information about your appointments and INR tests that are coming up. The information that I have says that you

were asked to check an INR and call your doctor on __________(Date)when you left the hospital.

- **CALLER:**
  - Can you please tell me when is the next INR scheduled? Does the patient know when their next INR was scheduled? ❑ Yes ❑ No
  - Who will you call with the INR result? Does the patient know who to call with the INR result? ❑ Yes ❑ No
  - When is your next appointment with the prescriber? Does the patient know when their next appointment is with the prescriber? ❑ Yes ❑ No
  - Are you going to be able to make it to do your INR test and to your appointment? Is there anything that might get in the way of your getting to these appointments? Did the patient express that were anticipating anything that might get in the way of going to the appointments? ❑ Yes ❑ No
    - **If yes, CALLER:** Let's talk about how we can work around these difficulties.
    - **If patient plans to keep appointment, ask, CALLER:** Do you have the hospital phone number to call the clinic (01-200800) if something unexpectedly comes up and you can't make the appointment?
    - **If patient can't keep appointment,** get the patient to reschedule: As soon as we hang up, can you call to reschedule your appointment?
    - Potential barriers to attendance identified: ❑ Yes ❑ No
    - List: ____
    - Potential solutions/resources identified: ❑ Yes ❑ No
    - List: ____
    - Alternative plan made: ❑ Yes ❑ No
    - Details: ____
    - Physician informed: ❑ Yes ❑ No
    - Details: ____

D. Coordination of Post Discharge Home Services (If noted that the patient will have INR test drawn at home):

- **CALLER:** I understand that you were going to have someone come home to check the INR. Will they still be coming to check the INR for you? ❑ Yes ❑ No
  - If no, **CALLER:** how will you be checking your INR?________________________

- Investigator or designee notified physician of the change in INR plans?
  ❑ Yes
  ❑ No
  ❑ No, Patient shared that there was a problem with home care providing services so there was a change in plan since discharge and the physician is aware.

F. Additional Notes

G. Time

- o Time for reviewing information prior to phone call: ________________________
- o Time for missed calls/attempts: ________________________
- o Time for initial phone call: ________________________
- o Time for talking to other health care providers: ________________________
- o Time for follow-up/subsequent phone calls to patient:________________________
- o Time for speaking with family or caregivers:________________________
- o Total time spent: ________________________

- o Caller’s Signature: ________________________
