# Supplementary material for: Impact of pharmacist-conducted anticoagulation patient education and telephone follow-up on transitions of care: a randomized controlled trial
Source: BMC Health Serv Res. 2021 Feb 16;21:151. doi: 10.1186/s12913-021-06156-2 (PMC7885504; doi:10.1186/s12913-021-06156-2)
Supplement: Supplementary file 3 — Additional file 3. Script for follow-up telephone call: 30 days post education. Script used at day 30 to assess readmissions, contact with health care providers or any bleeding event. [file 12913_2021_6156_MOESM3_ESM.pdf]

Appendix 3.
Script for follow-up telephone call: 30 days post education to ask about readmissions, contact with health care providers or any bleeding event

Hello, Can I please speak with ______________________ (name of study subject or whoever signed the consent form). This is Dr. ________________ calling you as a follow up to the study that you approved to be involved in during your stay at LAUMCRH. As you know, we shared with you that we would call you at 30 days after you received the education to ask you if you had any readmissions, contact with health care providers or any bleeding event . I just wanted to ask you a few questions.

- Since your hospital stay, have you needed to return to the hospital within 30 days after leaving the hospital on ____________(date)? □Yes □No
    - If yes, how many days after leaving the hospital did you have to return to the hospital?
    - If yes, how many times did you have to go back to the hospital?____(# of readmissions)

- Have you seen any healthcare provider (physician, nurse or pharmacist) pertaining to a concern or adverse effect to your blood thinner treatment within 30 days post discharge? □ Yes □ No
    - If yes, how many days after leaving the hospital did you have to see a health care professional?___
    - If yes, how many times did you have to see a healthcare professional?___(# of readmissions)

• **Did you have any of these problems**	**Yes**	**No**
Blood from your nose	☐	☐
Blood from your gum	☐	☐
Blood or redness in your eyes	☐	☐
Blood in the sputum	☐	☐
Purple /greenish bruises on your body	☐	☐
Blood in vomitus	☐	☐
Blood in stools	☐	☐
Dark black stools	☐	☐
Blood in urine	☐	☐
Any major bleeding such as in head, stomach, did you have to receive blood?	☐	☐

Bleeding ☐ ☐

If yes, ask the patient: When did you experience bleeding event?

Days post discharge________________

Were you receiving any blood thinners at the time of the bleed? ☐ ☐
